# Cross sectional study of drug substitution in community pharmacies in the Ugandan capital city

**DOI:** 10.1186/1472-6963-14-S2-P84

**Published:** 2014-07-07

**Authors:** Asha Nabbale, Richard Odoi-Adome, Freddy Kitutu

**Affiliations:** 1 Pharmacy Department, International Hospital of Kampala, Kampala, Uganda; 2 Pharmacy Department, Makerere University College of Health Sciences, Kampala, Uganda

## Background

The escalating cost of pharmaceuticals is a global challenge and major hindrance to access to medicines in developing countries. Half of the Ugandan population lacks reliable access to essential medicines and out of pocket expenditure by patients is as high as 80% [1]. Generic medicines provide an opportunity for savings on medicine expenditure due to their cheaper price [2,3]. Generic substitution has been recommended by World Health Organisation and is widely practised in Africa as long as the prescriber does not forbid nor the patient decline [4]. Therefore, this study was conducted to determine the nature and prevalence of drug substitution in community pharmacies in Kampala, the capital city of Uganda. Dispensers’ perceptions were also explored.

## Materials and methods

It was a cross sectional descriptive study employing two data collection tools; a structured questionnaire administered to dispensers in a random sample of community pharmacies and simulated patients presenting with prescriptions developed and validated by the research team.

## Results

Up to 133 community pharmacies in Kampala city were included in the study. Almost all (n=127, 96%) community pharmacies practised drug substitution. The most common forms of drug substitution were innovator medicine to generic medicine (85%) and generic medicine to other generic medicine (82%). Up to 92% of the pharmacies substitute “over the counter” drugs while 56% substitute medicines on prescription. Only 24% of the pharmacies did not consult the prescriber before drug substitution and majority (75%) considered the price of the drug before drug substitution. Knowledge of drug substitution policy was low and many (61%) dispensers thought Uganda has no national policy on drug substitution.

## Conclusion

Drug substitution involving both innovator medicine to generic medicine and generic to generic medicines is wide spread in community pharmacies in Kampala city.

The drug regulatory authority should focus on therapeutic equivalence studies and safety profile of approved generic products to better protect the public from substandard medicines.
